# Management and outcome of traumatic brain injury patients at Muhimbili Orthopaedic Institute Dar es Salaam, Tanzania

**DOI:** 10.11604/pamj.2017.26.140.10345

**Published:** 2017-03-14

**Authors:** Respicious Boniface, Edwin Rwebugisa Lugazia, Abel Mussa Ntungi, Othman Kiloloma

**Affiliations:** 1 Muhimbili Orthopaedic Institute (MOI), Dar es Salaam, Tanzania; 2 Injury Control Centre Tanzania (ICCT); 3 Muhimbili University of Health and Allied Sciences (MUHAS), Dar es Salaam, Tanzania; 4 Jakaya Kikwete Cardiac Institute, Dar es Salaam, Tanzania

**Keywords:** Traumatic brain injury, road traffic crashes, Tanzania

## Abstract

**Introduction:**

Traumatic brain Injuries represents a significant cause of morbidity and mortality worldwide and road traffic crashes accounts for a significant proportion of these injuries. However, access to neurosurgical care is poor in low income countries like Tanzania. The aim of this study was to assess the management and outcome of Traumatic brain injury patients at a tertiary level health facility in Tanzania.

**Methods:**

A retrospective observational study of Traumatic brain injury patients attended at Muhimbili Orthopedic Institute between January 2014 and June 2014.

**Results:**

A total of 627 Traumatic brain injury (TBI) patients were seen, 86% were males. Majority (73%) were between 15 - 45 years age group. Road traffic crashes were the leading cause of injury (59.3%). Majority 401/627 (64%) sustained mild TBI, 114/627 (18.2%) moderate TBI and 112/627 (17.8%) severe TBI. All mild TBI patients had good recovery. Among patients with moderate and severe TBI; 19.1% had good recovery, 50.2% recovered with disabilities and 30.7% died. Independent factors associated with mortality were: Severe TBI (Odds Ratio (OR) 3.16. 95%CI 3.42-10.52) and Systolic blood pressure at referring hospital of more than 90mmHg (Odds Ratio (OR) 0.13, 95%CI 0.04-0.49).

**Conclusion:**

Traumatic brain injury is a public health problem in Tanzania, mostly due to road traffic crashes. It is therefore important to reinforce preventive measures for road traffic crashes. There is also a need to develop and implement protocols for pre-hospital as well as in-hospital management of brain trauma in Tanzania.

## Introduction

Traumatic brain injury (TBI) is a major public health and socio - economic problem throughout the world. It is one of the leading causes of death, especially among young adults, and lifelong disability is common in those who survive [1]. Hence, exerting undue financial burden on governments and individuals in terms of treatment costs and lost manpower. It is estimated that in the USA, around 5.3 million people are living with a TBI related disability [2], and in the European Union 7.7 million people who have experienced a TBI have disabilities [3]. The World Health Organization estimates that each year more than 10 million people in the world sustain TBIs resulting in death or extensive hospitalization [4]. Most recent data indicate that TBI is responsible for more than 4.5 million deaths a year which translates to approximately one in every 10 deaths in the world. This number is expected to increase, especially due to the rapidly increasing rates of TBI in developing countries. It is for this reason that TBI has often been referred to as “the silent epidemic” [5], due to the fact that most of its consequences, especially cognitive impairments, are not obvious. The incidence of TBI worldwide is rising, mainly owing to injuries associated with the increased use of motor vehicles, particularly in middle-income and low-income countries [6]. Road traffic accidents are responsible for about 60% of brain injuries in the world. Globally, road traffic accidents are responsible for more than 50 million injuries every year, with about 1.2 million ending in death [7]. Other causes are falls, which account for about 25%, and other non-motor-vehicle-related accidents and acts of violence which collectively account for about 15% of TBIs [4].

As one of the rapidly developing nations in Africa, Tanzania has a significantly high rate of traffic-related deaths and disabilities most of which result from brain injuries. A hospital based injury surveillance involving six public hospitals in Tanzania, between November 2011 and December 2012 [8], revealed road traffic crashes to be the leading cause of injuries accounting for 47.5% of all injuries seen and 60.5% of injuries mortality. The risk of being killed in a traffic crash in Tanzania proportionate to the number of vehicles on the road is 20-30 times higher than in the USA and many countries in Western Europe [9], Different from the west, however, is the fact that pre-hospital care is almost nonexistent and health care service deliveries are poor. Police, good samaritans or relatives are frequently responsible for transport to healthcare facilities. Efforts to improve transport and trauma management in Tanzania will almost certainly result in improved patient outcomes. From pre-hospital to emergency department and ICU, a simultaneous assessment, monitoring, stabilization and therapeutic intervention of hypoxia and hypotension is important since a single episode of hypotension increases the risk of disability and death. The Brain Trauma Foundation developed the first TBI Guidelines in 1995 with the assistance of a group of international experts in the field. The goal was to offer the latest research on which to build protocols that would improve the survival and outcomes of TBI patients [10]. There has been some evidence that treatment in centers with neurosurgical support, especially in settings where protocol-driven neuro-intensive care units operate based on the above-referenced guidelines, is associated with better patient outcomes [11, 12]. This study was therefore conducted to help us understand the current handling of TBI patients at a tertially level health facility in Tanzania. The result of this study will help in developing guidelines to better assess, treat and monitor TBI patients with a view of improving patient outcomes.

## Methods

*Study Setting*: data were collected from Muhimbili Orthopedic Institute (MOI), which is a national trauma referral hospital. It is also a teaching hospital in the field of Orthopedic, Neurosurgery and Traumatology for Muhimbili University of Health and Allied Sciences. It is the main government trauma referral hospital serving the people of Dar es Salaam capital city of Tanzania where the hospital is located, also referred patients from other hospitals in the country.

*Study design*: a retrospective observational study was conducted

*Study population*: the study population comprised of traumatic brain injury patients attended at MOI between January 2014 and June 2014.

*Data collection and management*: a structured questionnaire was used by trained research assistants to extract data from patients' medical records. Data were abstracted on the following variables: socio-demographic factors, cause of injury, time from injury to MOI arrival, interventions done at referring hospital, interventions at Muhimbili Orthopedic Institute, severity (Glasgow coma scale) and patients' outcome (Death, recovery). Data were double-entered into Excel (MicrosoftÂ^®^ Excel, Seattle Washington), Statistical analysis was conducted using STATA version 11 (Statacorp, College Station, USA).

*Statistical analysis*: categorical variables were described using frequency distribution, mean and standard deviation for continuous variables. Chi-square test was used for bivariate analysis and those variables with observed frequency less than five Fisher's exact test was applied. A variable with (p ≤ 0.05) with mortality was considered to be statistically significant. Variables that demonstrated significant bivariate association with mortality were entered into the multivariate logistic regression modal to assess independent effects. Parameter of measurement to assess association was odds ratio.

*Ethical consideration*: ethical approval for the study was obtained from the Muhimbili University of Health and Allied Sciences Research Ethics Committee. Individual informed consent was not undertaken as the study used patients' medical records.

## Results

In a six months' study period 986 TBI patients were identified from patients register. 359 patients were excluded for the following reasons: 224 patients' medical records were missing and there was incomplete documentation for 135 patients. Thus, a total of 627 patients were included in the study. The age of patient varied from 1 year to 79 years with a mean age of 32.4 years. Majority (73%) belonged to age group of (15 to 45years), and 86% were males, with a male to female ratio of 6:1. Of the patients seen 518 (82.6%) arrived as referral patients. Road traffic crashes (59.3%) were responsible for the majority of TBIs, followed by Assault (24%), falls (12.7%) and others (4%). Motorcycles accounted for 51% of road traffic crashes, Car (43%) and Tricycles (6%). Majority 401/627 (64%) sustained mild TBI, 114/627 (18.2%) moderate TBI and 112/627 (17.8%) severe TBI.

### Management

#### Referring hospital

Management information at referral health facilities is summarized in Table 1. Majority (93%) were kept on room air ventilation. Oxygen saturation was not monitored in almost all patients (89.8%), and almost half (48.9%) had their Glasgow Coma Score not assessed. Neck stabilization was not performed in almost all patients (94.6%),

**Table 1:** Management at referral health facilities

Management	Number N	Percentage (%)
**Airway management**		
Mask/Nasal prongs	11	5.9
Intubated	2	1.1
Room air	173	93
Total	186	100
**Intravenous Fluids**		
Dextrose fluids	1	0.5
Normal saline/Ringers	124	66.7
None	61	22.8
Total	186	100
**Neck stabilization**		
Yes	10	5.4
No	176	94.6
Total	186	100
**Oxygen saturation**		
< 90%	3	1.6
>90%	16	8.6
Not monitored	167	89.8
Total	186	100
**Systolic blood pressure**		
< 90mmHg	17	9.1
> 90mmHg	98	52.7
Not monitored	71	38.2
Total	186	100
**GCS**		
Assessed	95	51.1
Not Assessed	91	48.9
Total	186	100

NB: GSC is Glasgow Coma Score

#### Muhimbili Orthopedic Institute (MOI)

Among patients with mild TBI, 152/401 (37.9%) patients were discharged on the same day of hospital arrival, while others were discharged after 24 hours of hospital admission and observation. Counseling on symptoms and signs of increasing intracranial pressure, altered level of consciousness or convulsion was done if that patient was to be discharged. Management information among patients with moderate and severe TBI at MOI is summarized in Table 2. Majority (85.6%) had CT scan (brain) done, 36.4% had brain contusion, 24.6% Epidural hematoma, 12.7% Subdural hematoma, 7.6% Skull fracture and 18.6% Intracerebral hemorrhage. Ninety percent of those with Epidural/Subdural hematoma had surgery, and time to surgery was within 6 hours from MOI arrival in 55.6% of patients, 19.1% patients had good recovery, and 88% of those with good recovery had moderate TBI, 50.2% recovered with disabilities and 30.7% died, 78.3% of those who died had severe TBI.

**Table 2:** Management at MOI

Management	Moderate injury N (%)	Severe injury N (%)	P-value
**CT Scan (brain)**			0.584
Yes	92 (57.5)	68 (42.5)	
No	14 (51.8)	13 (48.2)	
**CT Pathology**			0.001
Epidural Haematoma	20 (68.9)	9 (31.1)	
Subdural Haematoma	6 (40)	9 (60)	
Intracerebral Haemorrhage	5 (65.1)	17 (77.3)	
Contusion	28 (65.1)	15 (34.9)	
Skull Fracture	2 (22.2)	7 (77.8)	
**Mannitol**			0.631
Yes	20 (54.1)	17 (45.9)	
No	94 (49.7)	95 (50.3)	
**Antiseizures**			0.181
Yes	110 (49.8)	111 (50.2)	
No	4 (80.0)	1 (20.0)	
**Analgesia**			0.014
Yes	108 (49.1)	112 (50.9)	
No	6 (100.0)	0 (0.0)	
**Sedatives**			0.000
Yes	17 (16.2)	88 (83.8)	
No	97 (80.2)	24 (19.8)	
**Steroids**			0.986
Yes	2 (50.0)	2 (50.0)	
No	112 (50.5)	110 (49.6)	
**Ulcer prophylaxis**			0.000
Yes	42 (35.0)	78 (65.0)	
No	72 (67.9)	34 (32.1)	
**Anti-thrombosis**			0.000
Yes	13 (25.5)	38 (74.5)	
No	101 (57.7)	74 (42.3)	
**Surgery**			0.609
Yes	29 (53.7)	25 (46.3)	
No	82 (49.7)	83 (50.3)	
**Time to Surgery**			0.486
< 6 hours	14 (46.7)	16 (53.3)	
6 – 12 hours	9 (60.0)	6 (40.0)	
> 6 hours	6 (66.7)	3 (33.3)	
**Outcomes**			0.000
Good recovery	38 (88.4)	5 (11.6)	
Disability	61 (53.9)	52 (46.0)	
Death	15 (21.7)	54 (78.3)	

#### Factors associated with mortality

The proportional of patients, who experienced mortality was higher among those who had intracerebral haemorrhage 38.1%, followed by those with skull fracture 21.4% and subdural haematoma 16.7%. Patients of age group15 years to 45 years experienced higher level of mortality 71.8% compared to other age group, as did patients who had tracheal intubation 66.7% (Table 3). The Odds of death in patients who had Systolic blood pressure of above 90mmHg at referring hospital were 87% less than those who had Systolic blood pressure of less than 90mmHg. The risk of death was 3.16 times greater in those with severe TBI than those with moderate TBI.

**Table 3:** Factors associated with mortality

Factor	Death N (%)	No death N (%)	P value	Multivariate OR (95% CI)	P value
**Referring hospital Systolic Blood Pressure**			0.000++		
< 90 mmHg	13 (21.3)	4 (3.2)		1	
> 90mmHg	33 (54.1)	65 (51.0)		0.13 (0.04 – 0.49)	0.002++
Not Monitored	15 (24.6)	56 (44.8)		0.09 (0.03 – 0.39)	0.001++
Total	61(100)	125 (100)			
**Severity**			0.000++		
Moderate	15 (21.1)	99 (63.9)		1	
Severe	56 (78.9)	56 (36.1)		3.16 (3.42 – 10.52)	0.041++
Total	71(100)	155 (100)			
**CT Pathology**			0.000++		
Epidural Hematoma	25 (32.9)	4 (9.5)			
Subdural Hematoma	8 (10.5)	7 (16.7)			
Intracerebral Hemorrhage	6 (7.9)	16 (38.1)			
Contusion	37(48.7)	6 (14.3)			
Skull Fracture	0 (0.0)	9 (21.4)			
Total	76 (100)	42 (100)			
**Age (Years)**			0.045++		
< 15	5 (7.0)	22 (14.2)			
15 – 45	51 (71.8)	115 (74.2)			
> 45	15 (21.1)	18 (11.6)			
Total	71 (100)	155 (100)			
**Airway management**			0.000++		
Mask/Nasal prongs	17 (23.9)	104 (67.1)		1	
Intubated/Tracheostomy	54 (76.1)	51 (32.9)		2.25 (0.49 – 10.24)	0.291
Total	71 (100)	155 (100)			
**Sedatives**			0.000++		
Yes	49 (69.1)	56 (36.1)		1	
No	22 (30.9)	99 (63.9)		1.35 (0.40 – 4.54)	0.625
Total	71 (100)	155 (100)			
**Ulcer prophylaxis**			0.000++		
Yes	52 (73.2)	68 (43.9)		1	
No	19 (26.8)	87 (56.1)		0.44 (0.19 – 0.99)	0.05++
Total	71 (100)	155 (100)			
**Anti thrombosis**			0.035++		
Yes	21(29.6)	30 (19.4)			
No	50 (70.4)	125 (80.6)			
Total	71 (100)	155 (100)			

++ P value ≤0.05; N = number; OR = Odds ratio; CI = Confidence interval

## Discussion

This study confirms a high and continually increasing prevalence of TBI in Tanzania, it also revealed prominent gender bias with males outnumbering females in as far as TBI as well as fatal outcomes. This is expected, since most of the cases are due to Road traffic crashes, and the increased participation of young males in high risk activities like boarding and disembarking from moving city public buses. Similar to what has been observed elsewhere [13, 14], majority of the patients in this study were young individuals in the economically active age group 15 to 45 years), and this is a significant setback to family and society. Hence, a need for urgent public policy response with special reference to prevention and emergency care of road traffic crash victims.

Mortality among patients with severe TBI in this study was 78.2%. This is similar to other low income countries [15], but notably high compared to centers where standardized management protocols of pre-hospital and in hospital care are being followed, where reported mortality rate is 33% [14]. Resuscitation of head injured patient at the accident site is paramount in minimizing morbidity and mortality. This can be achieved through pre-hospital care which is non-existent in Tanzania. Targeted resuscitation and early specialist management have resulted in a decline in mortality over the last few decades [16]. The priorities are to prevent hypoxia and hypotension, both common findings after trauma. Even a single episode of hypotension is associated with increased morbidity and a doubling of mortality [17], as revealed in this study; patients who had a systolic blood pressure of less than 90mmHg at referring hospital had a greater Odd of mortality than patients who had systolic blood pressure of above 90mmHg. It is also noted in this study that at referring hospitals oxygen saturation was not monitored in almost all patients, this increases the chance of not detecting hypoxia, and hence increasing the risk of worse outcomes. There is therefore a need for intensive training of medical personnel's and adequate resources at primary and secondary level health facilities for establishing standardized care protocols. CT scan is the imaging modality recommended for assessment of intracranial pathology in patients with TBI [18, 19]. In this study however, no patient had CT scan done at referring hospital. 13.2% of those with moderate TBI and 16.4% of those with severe TBI at Muhimbili Orthopedic Institute didn't do CT scan. This is probably due to the great cost of CT scan, because in government health facilities of Dar es Salaam, CT scan is only available at Muhimbili National Hospital. There is a need for our government to invest more in CT scanners as the use of CT scan provides for optimal management.

In this study patients with severe TBI had increased Odds of mortality, this is similar to what has been reported in other studies elsewhere [20]. TBI patient's survival is highly dependent on timely and accurate management including appropriate nursing care [21, 22]. In our setup, we have nurses trained in ICU care, but it would be useful to train more nurses in neurosurgical care if the quality of observations is to be improved. In the absence of specially trained nurses and doctors, subtle neurological cues may be missed with consequent poor management. Intracranial pressure (ICP) monitoring is also indicated in patients with severe TBI patients [23]. The main objective of ICP monitoring is to maintain adequate cerebral perfusion and oxygenation and avoid secondary injury while the brain recovers. ICP monitoring is not done at Muhimbili Orthopedic Institute, and this may contribute to observed mortality among severe TBI patients in this study. There is therefore a need for management improvement strategies in order to improve outcome of TBI patients in Tanzania, including development and enforcement of evidence based TBI management protocols. In considering the findings of this study it is important to bear in mind the following limitations: firstly, this was a single centre study with small number of patients, and the time frame of the study was short, hence it may not reflect what is happening in other centers. Secondly, data collectors may not have collected all the data and so some are missing. Thirdly, challenges of collecting complete dataset and information bias from data collectors may have affected the quality of data.

## Conclusion

The results of this study provide valuable insight into the burden and management of TBI in Tanzania hospitals. It also reveals a low quality of care for patients with TBI in our setup. There is a need for a multidisciplinary team consisting of clinicians, researchers and policy makers to develop and implement evidence based protocols for pre-hospital as well as in-hospital management of brain trauma in Tanzania. Since most of the TBI cases are due to Road traffic crashes, there is a need for appropriate preventive measures to help reduce the frequency of head injury in our population. Public awareness campaigns concerning road safety rules are needed to help reduce the occurrence of road traffic crashes as well as improvement of roads.

### What is known about this topic

- Road traffic crashes account for much of the TBI burden in Tanzania;
- Majority of the injured victims are in the economically active age group of 15 - 45 years;
- Most of the victims are males.

### What this study adds

- This study reveals a low quality of care of patients with TBI in Tanzania hospitals;
- It also reveals lack of protocols for pre-hospital as well as in-hospital management of brain trauma in Tanzania.
